# Association of blood pressure with development of metabolic syndrome components: a five-year Retrospective Cohort study in Beijing

**DOI:** 10.1186/1471-2458-13-912

**Published:** 2013-10-02

**Authors:** Da Huo, Lixin Tao, Xia Li, Wei Wang, Zhaoping Wang, Dongning Chen, Huiping Zhu, Xinghua Yang, Yanxia Luo, Xiuhua Guo

**Affiliations:** 1 School of Public Health, Capital Medical University, No. 10 Xitoutiao, You’anmen Wai, Fengtai District, Beijing 100069, China; 2 Beijing Municipal Key Laboratory of Clinical Epidemiology, Beijing 100069, China; 3 Institute for Infectious Disease and Endemic Disease Control, Beijing Center for Disease Prevention and Control, No. 16 Hepingli Middle Street, Dongcheng District, Beijing 100013, China; 4 Department of Epidemiology and Public Health, University College Cork, Fourth Floor, Western Gate Building, Cork, Ireland; 5 Department of Integrated Early Childhood Development, Capital Institute of Pediatrics, No. 2 Yabao Road, Chaoyang District, Beijing 100020, China; 6 Physical Examination Department, Beijing Tongren Hospital, Capital Medical University, No. 1 Dongjiao Minxiang, Dongcheng District, Beijing 100730, China

**Keywords:** Metabolic syndrome, Blood pressure, Retrospective cohort study, Chinese

## Abstract

**Background:**

Raised blood pressure (BP) is associated with the incidence of metabolic syndrome (MetS). It is unknown if subjects with different BP levels may develop certain components of MetS over time. We investigated the incidence of MetS relative to different levels of BP over a 5-year period in a Chinese population in Tongren Hospital, Beijing.

**Methods:**

During the period of 2006–2011, we recruited 2,781 participants with no MetS, or self-reported type 2 diabetes, dyslipidemia, hypertension, or cardiovascular disease at baseline. Association rule was used to identify the transitions of MetS components over time.

**Results:**

The incidence of MetS at follow-up was 9.74% for men and 3.21% for women in the group with optimal BP; 10.29% and 7.22%, respectively, in the group with normal BP; 10.49% and 10.84%, respectively, in the group with high-normal BP; and 14.48% and 23.21%, respectively in the group with high BP. The most common transition was from healthy to healthy in the groups with optimal or normal BP (17.9–49.3%), whereas in the high-normal BP group, 16.9-22.1% of subjects with raised BP returned to healthy status or stayed unchanged, while 13.8-21.4% of people with high BP tended to develop raised fasting glucose levels.

**Conclusions:**

The incidence of MetS increased in parallel with the increase in BP. People with optimal and normal BP levels were less susceptible to developing MetS over time, whereas abnormal BP seemed to be a pre-existing phase of MetS. High-normal BP was a crucial status for MetS prevention.

## Background

Metabolic syndrome (MetS) is a complex medical condition comprising five inter-related risk factors within an individual: obesity, hypertension, hypertriglyceridemia, low high-density lipoprotein cholesterol (HDL-C), and hyperglycemia [1,2]. It is a growing public-health issue and with approximately one quarter of the world adult population having the disorder [3]. Several studies have indicated that insulin resistance is a major underlying cause of MetS [4-6]. And MetS is frequently reported to be in association with cardiovascular disease (CVD) [7-10]. But the clinical value of MetS and its effect on a patient life is still controversial [11,12]. As a result, there is an absence of conclusive recommendations for patients with MetS [13].

Hypertension may trigger the pathogenesis of CVD, and has been shown to be a key factor of MetS [14]. The Framingham Heart Study showed that hypertension played a central role in MetS, and was the most commonly associated risk factor for MetS [15]. Subjects with certain combinations of MetS components showed greater arterial changes, and MetS might be a consequence of raised blood pressure (BP). Thus, MetS predicts the onset of CVD, as it has deleterious effects associated with central arterial aging [16,17].

Although these results may provide clues as to which risk factors underlie the pathophysiology of MetS, there have been few studies investigating which MetS components tend to cluster dominantly, and how the state of each risk factor changes over time [15,18].

In the present study, we classified subjects with different levels of BP into four groups at baseline in order to investigate: 1) whether there is a correlation between the level of BP and the incidence of MetS; 2) what risk factors tend to develop at each level of BP; and 3) how these risk factors change with time.

## Methods

### Survey methodology and laboratory tests

At Beijing Tongren Hospital, subjects’ health records of routine physical check-ups have been computerized since 2006. We selected the records of adult visitors who had a physical check-up, using in 2006/2007 as the baseline, and 2010/2011 as the end point. Totally, there were 7,512 people who had check-up records both at baseline and the follow-up. 3,406 subjects with missing data of weight, height, systolic or diastolic blood pressure (SBP, DBP), fasting blood glucose (FPG), triglycerides(TG), or HDL-C were excluded. 950 subjects with any medical history of stroke, heart failure, angina pectoris or myocardial infarction, or in use of any hypoglycemic, anti-dislipidemic, or antihypertensive treatment were excluded as well. 975 subjects who had MetS at baseline were ruled out. The final cohort was composed of 2,781 participants.

The physical check-ups included measurements of weight, height, and BP, and analyses of blood biochemistry parameters. Body mass index (BMI) was calculated as weight/height^2^ (kg/m^2^). Patients were given a five-minute rest in a seated position before systolic and diastolic blood pressure (SBP, DBP) were measured with a mercury sphygmomanometer.

The blood biochemical analyses included FPG and HDL-C. The blood samples were taken from the cubital vein in the morning after 8 o’clock. Each individual confirmed that there had been no food intake for least 10 hours. The samples were analyzed immediately after pre-treatment or stored at -80°C for further analysis. Serum HDL-C concentration was measured photometrically (Hitachi 704; Roche, Mannheim, Germany), and TG and FPG concentrations were determined enzymatically (Hitachi 717; Roche Diagnostics), with all analyses performed in accordance with the manufacturer’s recommendations.

### Definition of metabolic syndrome

Diagnostic criteria for the assessment of MetS components were defined according to the Joint Scientific Statement of MetS [19]. However, as waist circumference was not obtained, BMI was taken as a substitute for the component of obesity, as it was reported previously that BMI was strongly associated hypertension and CVD in a population of northern Chinese [20]. The diagnosis criteria used in our research were as follows:

•Overweight: BMI ≥ 25 and < 30 kg/m^2^; obese: BMI ≥ 30 kg/m^2^[21];

•Raised TG level (drug treatment for raised triglyceride level is an alternative indicator): ≥ 150 mg/dl (1.7 mmol/L) [19];

•Reduced HDL-C level (drug treatment for reduced HDL-C level is an alternative indicator): < 40 mg/dl (1.0 mmol/L) in male; < 50 mg/dl (1.3 mmol/L) in female [19];

•Raised BP (antihypertensive drug treatment in a patient with a history of hypertension is an alternate indicator): SBP ≥ 130 mmHg and/or DBP ≥ 85 mmHg [19];

•Raised FPG level (drug treatment of raised glucose is an alternative indicator): ≥ 100 mg/dL (5.6 mmol/L) [19];

Participants fulfilling at least three out of these five components were diagnosed as having MetS.

### Layered approach for blood pressure levels at baseline

Subjects were divided into subgroups according to their BP at entry time using the WHO classification criteria [22]. To avoid the overlap of “optimal pressure” and “normal pressure” in the WHO criteria, we defined optimal pressure as SBP < 120 and DBP < 80 mmHg; normal BP as SBP ≥120 and <130 mmHg, and/or DBP ≥80 and <85 mmHg; high-normal BP as SBP ≥130 and <140 mmHg, and/or DBP ≥85 and <90 mmHg; and high BP as either SBP ≥140 or DBP ≥90 mmHg. When a visitor’s SBP and DBP were in different categories, the higher category was used.

### Statistical analysis

Categorical data are presented as percentages, and continuous data as mean plus standard deviation (SD). The five individual risk factor states were analyzed at baseline and follow-up.

We performed association rule to analyze the changes in MetS component or their combinations during the five-year period in our cohort. As the subjects at baseline were people with two or less MetS components, there were 16 (i.e. $C_{5}^{0}+C_{5}^{1}+C_{5}^{2}$) possible states at baseline, with 32 (i.e. 2^5^) possible states at follow-up. Theoretically, there were 512 (i.e. 16 × 32) possible transitions between states from baseline to follow-up. The data are presented as transitions of states. The module of association rule in SAS software (version 9.1; SAS Institute, Chicago, IL, USA) was used to generate the change from a risk factor state to another, with “healthy” defined as being the state free of MetS component. Data preparation and descriptive statistics were using the same software.

### Ethics statement

This study was approved by the ethics committee of the Capital Medical University of China, and performed in accordance with the principles of Declaration of Helsinki (reference no. 2013SY26).

### Consent

Written informed consent was obtained from the patient for publication of this report and any accompanying images.

## Results

At baseline, of the 2,781 subject, 43.7% were mens with the median age of 36.0 years-old (39.0 ± 11.1, mean ± SD), while 56.3% were women with the median age of 39.0 years-old (39.3 ± 10.2). The follow-up interval was 4.83 ± 0.44 years for men and 4.76 ± 0.48 years for women, respectively. To adjust for confounding variables, gender, age, BMI, FPG, SBP, TG and HDL-C at baseline were included in binary logistic regression. The results are shown in Table 1.

**Table 1:** The results of logistic regression model

**Variable**	**B**	**S.E.**	**Wald**	**d.f.**	***P *value**	**OR**	**95% CI for OR**	
							**Lower**	**Upper**
Sex	−0.115	0.125	0.845	1	0.358	0.891	0.698	1.139
Age	0.026	0.005	25.670	1	<0.001	1.027	1.016	1.037
BMI	0.340	0.022	232.297	1	<0.001	1.406	1.345	1.468
FPG	0.060	0.081	0.542	1	0.461	1.062	0.905	1.246
SBP	0.024	0.004	32.247	1	<0.001	1.024	1.016	1.032
TG	0.447	0.072	38.876	1	<0.001	1.564	1.359	1.800
HDL-C	−1.412	0.219	41.701	1	<0.001	0.244	0.159	0.374
Constant	−12.010	0.857	196.490	1	<0.001	0.000		

**Figure 1:**
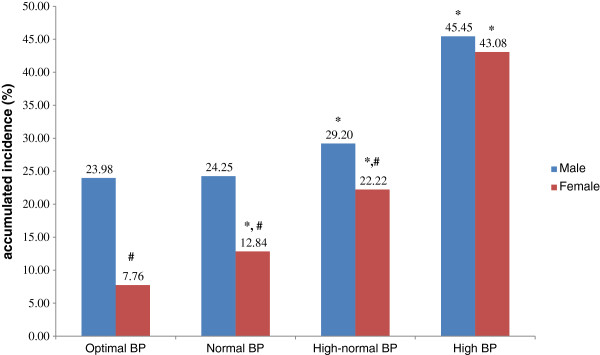
The accumulated incidence of MetS in subjects aged 18–49, stratified by gender and blood pressure level. ^*^Compared with optimal BP group of same gender using *χ*^2^ test, *P* < 0.001. ^#^Compared with male counterpart using *χ*^2^ test, *P* < 0.001.

**Figure 2:**
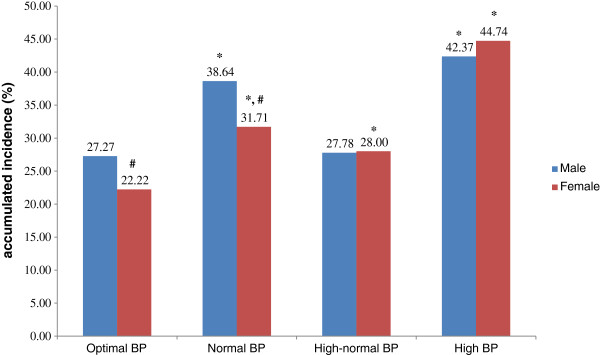
The accumulated incidence of MetS in subjects aged ≥50, stratified by gender and blood pressure level. ^*^Compared with optimal BP group of same gender using *χ*^2^ test, *P* < 0.001. ^#^Compared with male counterpart using *χ*^2^ test, *P* < 0.001.

The components of MetS in the population at baseline and follow-up were grouped by BP level at baseline (Tables 2, 3, 4 and 5). Subjects were divided in two age groups (18–49 and ≥50), which stands for young and old people. The incidence of MetS at follow-up was analyzed by gender and BP level at baseline (Figures 1 and 2). Young men with optimal, normal and high-normal BP had a higher incidence of MetS compared with women in the same groups (23.98% vs. 7.76%, 24.25% vs. 12.84% and 29.20% vs. 22.22%, respectively). The numbers of each gender were almost the same in the high BP group (45.45% vs. 43.08%). Whereas old men with optimal, normal and high-normal BP had a higher incidence of MetS compared with women in the same groups (27.27% vs. 22.22%, 38.64% vs. 31.71, respectively). The numbers of each gender were almost the same in the high-normal and high BP group (27.78% vs. 28.00%, 42.37% vs. 44.74).

**Table 2:** The profile of MetS components in male subjects aged 18–49 of different blood pressure group at baseline (n = 965)

**Characteristic**	**Optimal BP (*n *= 342)**			**Normal BP (*n *= 367)**			**High-normal BP (*n *= 113)**			**High BP (*n *= 66)**		
	**Baseline**	**Follow-up**	***P*-value**	**Baseline**	**Follow-up**	***P*-value**	**Baseline**	**Follow-up**	***P*-value**	**Baseline**	**Follow-up**	***P*-value**
BMI, kg/m^2^	23.46 ± 3.10	24.35 ± 3.15	<0.001	23.67 ± 2.83	24.44 ± 2.87	<0.001	24.10 ± 3.07	24.50 ± 2.93	0.001	24.94 ± 2.96	25.57 ± 3.22	<0.001
SBP, mmHg	103.70 ± 7.33	112.51 ±11.49	<0.001	116.04 ± 6.74	117.32 ±10.61	0.043	125.88 ± 7.05	121.82 ±12.04	0.001	131.85 ±12.76	127.611±15.06	0.001
DBP, mmHg	68.86 ± 4.42	74.61 ± 8.94	<0.001	77.41 ± 4.28	77.14 ± 8.04	0.557	82.26 ± 4.28	79.93 ±10.06	0.017	92.99 ± 7.10	85.30 ± 9.61	<0.001
HDL-C, mmol/L	1.34 ± 0.29	1.24 ± 0.29	<0.001	1.34 ± 0.27	1.27 ± 0.30	<0.001	1.38 ± 0.27	1.36 ± 0.31	0.295	1.35 ± 0.24	1.30 ± 0.28	0.003
TG, mmol/L	1.36 ± 1.17	1.61 ± 1.37	<0.001	1.28 ± 0.73	1.64 ± 1.61	<0.001	1.33 ± 0.98	1.47 ± 0.99	0.084	1.39 ± 0.97	1.60 ± 1.03	0.003
FPG, mmol/L	5.12 ± 0.46	5.24 ± 0.53	<0.001	5.26 ± 0.76	5.37 ± 0.82	0.006	5.28 ± 0.53	5.34 ± 0.81	0.279	5.37 ± 0.68	5.67 ± 1.55	0.007

*BMI* body mass index, *SBP, DBP* systolic / diastolic blood pressure, *HDL-C* high-density lipoprotein cholesterol, *TG* triglyceride, *FPG* fasting plasma glucose. *P*-value is based on paired *t*-test.

**Table 3:** The profile of MetS components in male subjects aged 50 and above with different blood pressure group at baseline (n = 249)

**Characteristic**	**Optimal BP (*n *= 66)**			**Normal BP (*n *= 88)**			**High-normal BP (*n *= 36)**			**High BP (*n *= 59)**		
	**Baseline**	**Follow-up**	***P*-value**	**Baseline**	**Follow-up**	***P*-value**	**Baseline**	**Follow-up**	***P*-value**	**Baseline**	**Follow-up**	***P*-value**
BMI, kg/m^2^	24.42 ± 3.16	24.49 ± 3.24	0.753	24.57 ± 2.57	24.88 ± 2.73	0.008	23.57 ± 2.59	24.02 ± 2.67	0.015	24.03 ± 2.85	24.44 ± 3.19	0.004
SBP, mmHg	103.11 ± 7.33	111.71 ±14.93	<0.001	115.23 ± 6.99	121.38 ±14.05	<0.001	127.50 ± 5.67	130.28 ±11.93	0.151	132.88 ±14.72	127.14 ±15.03	0.004
DBP, mmHg	68.86 ± 4.87	74.05 ±10.11	<0.001	77.50 ± 4.29	80.17 ±10.67	0.025	80.97 ± 4.44	80.25 ±10.82	0.665	89.07 ± 7.74	83.24 ± 7.87	<0.001
HDL-C, mmol/L	1.42 ± 0.28	1.35 ± 0.29	0.003	1.34 ± 0.31	1.29 ± 0.35	0.097	1.45 ± 0.28	1.38 ± 0.31	0.060	1.46 ± 0.37	1.39 ± 0.33	0.036
TG, mmol/L	1.43 ± 0.98	1.43 ± 0.77	0.998	1.42 ± 0.87	1.60 ± 1.35	0.118	1.33 ± 0.75	1.39 ± 0.72	0.618	1.34 ± 0.80	1.42 ± 0.74	0.396
FPG, mmol/L	5.67 ± 1.21	5.72 ± 1.13	0.719	5.74 ± 1.00	5.88 ± 0.85	0.088	5.67 ± 1.32	5.64 ± 0.77	0.897	5.63 ± 0.94	5.83 ± 1.04	0.011

*BMI* body mass index, *SBP, DBP* systolic/diastolic blood pressure, *HDL-C* high-density lipoprotein cholesterol, *TG* triglyceride, *FPG* fasting plasma glucose. *P*-value is based on paired *t*-test.

**Table 4:** The profile of MetS components in female subjects aged 18–49 of different blood pressure group at baseline (n = 1 323)

**Characteristic**	**Optimal BP (*n *= 838)**			**Normal BP (*n *= 366)**			**High-normal BP (*n *= 54)**			**High BP (*n *= 65)**		
	**Baseline**	**Follow-up**	***P*-value**	**Baseline**	**Follow-up**	***P*-value**	**Baseline**	**Follow-up**	***P*-value**	**Baseline**	**Follow-up**	***P*-value**
BMI, kg/m^2^	21.48 ± 2.67	22.17 ± 2.83	<0.001	22.24 ± 2.71	22.83 ± 2.82	0.097	22.96 ± 2.63	23.63 ± 2.75	0.025	23.76 ± 2.36	24.27 ± 2.68	0.011
SBP, mmHg	101.03 ± 7.43	107.19 ±11.82	<0.001	115.48 ± 7.09	114.35 ±12.32	<0.001	124.63 ± 7.32	119.80 ±14.39	0.025	129.00 ±14.66	126.88 ±16.51	0.265
DBP, mmHg	66.08 ± 5.12	70.23 ± 7.97	<0.001	77.79 ± 4.14	75.09 ± 8.39	<0.001	82.31 ± 3.46	79.98 ± 9.62	0.089	90.31 ± 8.00	83.55 ±11.48	<0.001
HDL-C, mmol/L	1.64 ± 0.33	1.58 ± 0.33	<0.001	1.62 ± 0.34	1.57 ± 0.38	<0.001	1.66 ± 0.27	1.51 ± 0.33	<0.001	1.62 ± 0.31	1.53 ± 0.35	0.010
TG, mmol/L	0.86 ± 0.48	0.98 ± 0.67	<0.001	1.03 ± 0.82	1.12 ± 0.83	0.014	1.02 ± 0.41	1.23.± 0.78	0.023	1.13 ± 0.60	1.32 ± 0.72	0.028
FPG, mmol/L	5.05 ± 0.44	5.12 ± 0.47	<0.001	5.20 ± 0.47	5.26 ± 0.64	0.039	5.30 ± 0.54	5.36 ± 0.71	0.324	5.22 ± 0.39	5.37 ± 0.67	0.066

*BMI* body mass index, *SBP, DBP* systolic/diastolic blood pressure, *HDL-C* high-density lipoprotein cholesterol, *TG* triglyceride, *FPG* fasting plasma glucose. *P*-value is based on paired *t*-test.

**Table 5:** The profile of MetS components in female subjects aged 50 and above of different blood pressure group at baseline (n = 244)

**Characteristic**	**Optimal BP (*n *= 99)**			**Normal BP (*n *= 82)**			**High-normal BP (*n *= 25)**			**High BP (*n *= 38)**		
	**Baseline**	**Follow-up**	***P*-value**	**Baseline**	**Follow-up**	***P*-value**	**Baseline**	**Follow-up**	***P*-value**	**Baseline**	**Follow-up**	***P*-value**
BMI, kg/m^2^	24.11 ± 2.06	23.43 ± 3.05	0.451	23.49 ± 2.46	23.52 ± 2.53	0.858	22.55 ± 2.29	23.10 ± 2.92	0.027	24.17 ± 3.18	24.09 ± 3.53	0.699
SBP, mmHg	103.23 ± 6.56	111.02 ±13.12	<0.001	116.46 ± 7.00	118.46 ±15.21	0.273	128.40 ± 4.73	124.44 ±12.93	0.143	139.34 ±15.03	134.76 ±19.41	0.149
DBP, mmHg	67.53 ± 4.76	71.66 ± 8.13	<0.001	78.17 ± 4.05	74.27 ± 8.71	<0.001	79.80 ± 5.30	77.20 ± 8.23	0.215	86.18 ± 9.11	80.16 ±11.85	0.002
HDL-C, mmol/L	1.64 ± 0.36	1.60 ± 0.38	0.060	1.63 ± 0.36	1.51 ± 0.32	<0.001	1.85 ± 0.35	1.68 ± 0.30	0.004	1.72 ± 0.28	1.54 ± 0.28	<0.001
TG, mmol/L	1.19 ± 0.70	1.35 ± 0.80	0.007	1.33 ± 1.07	1.49 ± 0.88	0.059	1.07 ± 0.34	1.25 ± 0.66	0.080	1.27 ± 0.58	1.34 ± 0.59	0.456
FPG, mmol/L	5.26 ± 0.53	5.27 ± 0.55	0.868	5.37 ± 0.53	5.42 ± 0.48	0.474	5.24 ± 0.44	5.43 ± 0.47	0.047	5.51 ± 0.47	5.65 ± 0.66	0.173

*BMI* body mass index, *SBP, DBP* systolic / diastolic blood pressure, *HDL-C* high-density lipoprotein cholesterol, *TG* triglyceride, *FPG* fasting plasma glucose. *P*-value is based on paired *t*-test.

**Figure 3:**
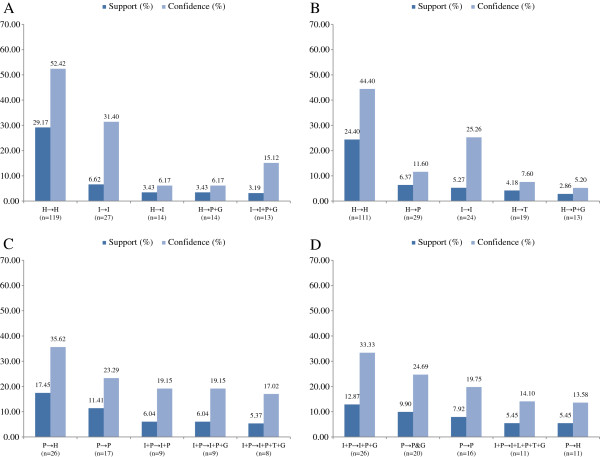
**The support and confidence rate of the top five transitions in female subjects from 2006/2007 to 2010/2011 in different blood pressure groups.** The support and confidence rate of the top five transitions in male subjects from 2006/2007 to 2010/2011 in different blood pressure groups. **(A)** in optimal BP group; **(B)** in normal BP group; **(C)** in normal-high BP group; **(D)** in high BP group. Abbreviations: H, health, with the absence of any MetS components; P, high blood pressure; G, high fasting plasma glucose; I, elevated body mass index; T, raised triglycerides level.

**Figure 4:**
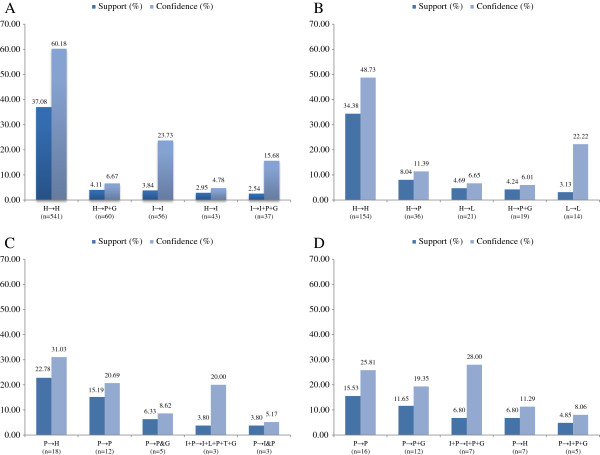
**The support and confidence rate of the top five transitions in male subjects from 2006/2007 to 2010/2011 in different blood pressure groups.** The support and confidence rate of the top five transitions in female subjects from 2006/2007 to 2010/2011 in different blood pressure groups. **(A)** in optimal BP group; **(B)** in normal BP group; **(C)** in normal-high BP group; **(D)** in high BP group. Abbreviations: H, health, with the absence of any MetS components; P, high blood pressure; G, high fasting plasma glucose; I, elevated body mass index; T, raised triglycerides level.

The five most common transitions in each subgroup were identified (Figures 3 and 4). As the sample size was too small after grouping by age. The association rule is applied without sub-stratification of age. The support rate of transitions (defined as the percentage of initial state to another state in all possible transitions) was examined in the period 2006/2007 to 2010/2011 in the different subgroups at baseline. For visual simplicity, the five most commonly observed transitions are shown in the parts of the relevant figures. The most common transition in both genders with optimal and normal BP was “healthy” to “healthy”, whereas for the high-normal BP group, the main transition was from “high BP” to “healthy”, and for the group with high BP, the major transition was from “high BP” to “high BP with high fasting glucose”. The rates of transition are shown for each gender and BP group. The confidence rate means how many cases transitioned within a certain status. For example, 244 male subjects were initially healthy at baseline, and 142 (53.79%) of these stayed healthy, while 23 (8.71%) transitioned to hypertension and high fasting glucose at the end of the observation period.

## Discussion

The present cohort study, comprising 2,781 subjects in Beijing Tongren Hospital, focused on detecting how the incidence of MetS changed relative to BP level and what MetS components tend to emerge and change during a 5-year follow-up period. Our study revealed that the higher the BP at baseline, the higher the incidence of MetS at a later stage. We identified that people with different levels of BP would develop different combinations of risk factors. On analyzing the change in risk factors, we found that people with high BP tended to have impaired fasting glucose as the most common additional and new-onset MetS component, and that people with a lower BP level developed raised BP as the initial risk factor of MetS.

SBP and DBP of people with optimal and normal blood pressure at baseline tended to increase with time, while the SBP or DBP dropped in the subjects with high-normal and high blood pressure, according to original Tables 1 and 2. People with high-normal and high blood pressure may be more alert to their health as health education and promotion programs are popular in varies media in Beijing, possible actions could be intentionally or unintentionally taken, and their SBP/ DBP is, therefore, controlled or even lowered.

An association between raised BP and MetS has been reported as the most common MetS component [23], or even serves as an independent predictor for CVD [24]. Several studies with structural equation modeling indicated that hypertension might play both a direct and indirect roles in the development of MetS [25-27]. However, physiologically, it is not easy to connect insulin-resistance with MetS [28]. In the present study, we found that people with optimal and normal BP tended to have a relative lower incidence of MetS after 5 years comparing with people with high BP, and were less susceptible to developing the disorder. Most subjects who started off as “healthy” remained “healthy”, similar to a previous study in a German population [29]. However, abnormal BP tended to be the first risk factor for MetS. About 17% of subjects with high-normal BP returned to “healthy”, while 12% continued to be in the condition.

By analyzing the shifts in MetS components, we found that raised BP was the most common risk factor for all groups. Previous studies have also shown that hypertension was the most important MetS component for men and one of the three most important components for women [30]. Previous study suggested that prehypertension (SBP = 120–139 mmHg, and/or DBP = 80–89 mmHg) was predictive for risk of MetS [31].

In our study, subjects with high BP tended to have impaired fasting plasma glucose as a secondary risk factor. This is accordance with other studies, which found that raised fasting glucose was the second common component of MetS and people with MetS tended to have a disorder of glucose metabolism [32]. Prehypertension may be an end-point related to each of the other four components. It is mainly a consequence of systemic low-grade inflammation and apoA-I dysfunction. In addition to the five components of MetS, prothrombotic and proinflammatory states are essential features based on the evidence of impaired function of HDL and apo A-I particles is discernible by biological evidence of functional defectiveness via outcomes studies and/or correlations with inflammatory and anti-inflammatory biomarkers [33]. The aggregation to lipoprotein (Lp)(a) of apolipoprotein (apo) A-I underlies HDL dysfunction, and is an independent risk factor of magnitude similar to conventional components of MetS [34]. Some studies indicated that proinflammatory state and oxidative stress are crucial for cardiometabolic disorders. Factors such as creatinine, platelet-activating factor, acetylhydrolase, thyroid stimulating hormone, acylation-stimulating protein, asymmetric dimethylarginine, and serum lipoprotein (Lp) (a) are key to trigger systemic low-grade inflammation and enhanced autoimmune reactions, which may induce impaired glucose and metabolic syndrome [35].

In most circumstances, “healthy” was the predominant state, and people with a single risk factor tended to return to the “healthy” state. High-normal BP was a crucial status for MetS prevention. As hypertension has a low rate of awareness in China, it is an important preventable risk factor for MetS and CVD events [36].

The strengths of this research were that it was a longitudinal study of 5 years in a Chinese population with data of relatively good quality. The limitation was the lack of waist circumference as an indicator for central obesity; however, we used BMI for substitute according to the WHO expert consultation [21]. As Tongren Hospital is located in the center of Beijing, bias may be that there were more people with an urban life style recruited for the research. In addition, this study was based on a population attending for routine health check-up. Further studies using the general population would be desirable.

## Conclusions

The incidence of MetS augments with the elevation of BP over time. People with optimal and normal BP were less susceptible than people with higher BP to developing MetS. Although most cases stayed “healthy”, they tended to have abnormal BP as the initial sign of MetS. High-normal BP was a crucial status for MetS prevention as some of the cases were able to return “healthy”. In the high BP group, high fasting glucose was a secondary risk factor. More efforts are needed to identify effective intervention for individuals with abnormal BP to prevent those developing MetS.

## Competing interests

The authors declare that they have no competing interests.

## Authors’ contributions

DH, LT, XL, and WW contributed to the analysis and interpretation of the data, drafting of the article, critical revision of the article, and statistical analysis; ZW, DC and XG contributed to the study concept and design, acquisition of data, field investigation and quality control; DH, LT, XY, YL and XG contributed to the drafting of the article, and critical revision of the article; XG had full access to all of the data in the study and takes responsibility for the integrity of the data and the accuracy of the data analysis. All authors gave their final approval for the manuscript.

## Pre-publication history

The pre-publication history for this paper can be accessed here:

http://www.biomedcentral.com/1471-2458/13/912/prepub
